# Transfer Learning Assisted Classification and Detection of Alzheimer’s Disease Stages Using 3D MRI Scans

**DOI:** 10.3390/s19112645

**Published:** 2019-06-11

**Authors:** Muazzam Maqsood, Faria Nazir, Umair Khan, Farhan Aadil, Habibullah Jamal, Irfan Mehmood, Oh-young Song

**Affiliations:** 1 Department of Computer Science, COMSATS University Islamabad, Attock Campus, Attock 43600, Pakistan; muazzam.maqsood@cuiatk.edu.pk (M.M.); umair_khan@cuiatk.edu.pk (U.K.); farhan.aadil@cuiatk.edu.pk (F.A.); 2 Department of Computer Science, Capital University of Science and Technology, Islamabad 45750, Pakistan; faria.nazir@cust.edu.pk; 3 Faculty of Engineering Sciences, Ghulam Ishaq Khan Institute, Topi 23460, Pakistan; habibullah@giki.edu.pk; 4 Department of Media Design and Technology, Faculty of Engineering & Informatics, University of Bradford; Bradford BD7 1DP, UK; irfanmehmood@ieee.org; 5 Department of Software, Sejong University, Seoul 05006, Korea

**Keywords:** Alzheimer’s Detection, AlexNet, ImageNet, transfer learning, contrast stretching, K-Mean clustering

## Abstract

Alzheimer’s disease effects human brain cells and results in dementia. The gradual deterioration of the brain cells results in disability of performing daily routine tasks. The treatment for this disease is still not mature enough. However, its early diagnosis may allow restraining the spread of disease. For early detection of Alzheimer’s through brain Magnetic Resonance Imaging (MRI), an automated detection and classification system needs to be developed that can detect and classify the subject having dementia. These systems also need not only to classify dementia patients but to also identify the four progressing stages of dementia. The proposed system works on an efficient technique of utilizing transfer learning to classify the images by fine-tuning a pre-trained convolutional network, AlexNet. The architecture is trained and tested over the pre-processed segmented (Grey Matter, White Matter, and Cerebral Spinal Fluid) and un-segmented images for both binary and multi-class classification. The performance of the proposed system is evaluated over Open Access Series of Imaging Studies (OASIS) dataset. The algorithm showed promising results by giving the best overall accuracy of 92.85% for multi-class classification of un-segmented images.

## 1. Introduction

Alzheimer’s disease (AD or Alzheimer’s) is a form of dementia mainly characterized by the incapacitation of the thought process and loss of the ability to conduct daily-routine tasks [1,2]. The process is a sedated degeneration of brain cells causing short-term memory loss and frequent behavioral concerns. The degenerative process occurs mainly in the ages of 60 and above. However, early diagnosis of Alzheimer’s disease has also been reported in subjects of ages 40 to 50 years. According to one estimation, about 5 million people in the United States are suffering from Alzheimer’s disease and with this rate, the number will be tripled by 2050 [3]. 

In this era of research and development, Alzheimer’s disease still lacks a distinctive treatment [4,5]. If diagnosed in a timely manner, its progressive patterns can be determined, affecting many lives of Alzheimer’s patients. Recent developments in neuro-imaging analysis have proven to be helpful in prior and accurate detection of AD. The neuro-imaging study utilizes MRI due to its high-level contrast along with spatial resolution and availability [6]. Various computer-assisted techniques are proposed for the computer-aided diagnosis of AD, classifying the characterized extracted features. These features are usually extracted from the regions of interest (ROI) and volume of interests (VoI) [7,8,9]. Similarly, the characteristic features were extracted from the varying Grey Matter (GM) voxels [2] and regions of the hippocampus [10,11,12]. These methods provide improved early diagnosis of Alzheimer’s disease. Most of the existing work has focused on the binary classification, which only shows whether someone has Alzheimer’s disease or not. However, proper treatment for Alzheimer’s needs a classification of a patient into no dementia, very mild, mild, and moderate dementia. This multiclass classification problem remains an open research area.

Massive progress has been made in the field of image processing, mainly because of the availability of large labeled datasets for better and accurate learning of models. Among those datasets is one of the most widely used datasets, ImageNet [13]. The dataset offers around 1.2 million natural images with above 1000 distinctive classes. Convolutional Neural Networks (CNN) trained over such datasets offer high accuracy and improvement in terms of medical imagery categorization. There are different techniques that can be used to utilize CNN for medical imagery classification [14,15,16,17]. Among them is the development of a pre-trained network over a large dataset followed by the fine-tuning of the model over the dataset to be classified. However, conventional image descriptors integrating low-level features have shown promising results. Fine tuning of a Convolutional Neural Network (CNN) using transfer learning serves as a significant alternative to training it from scratch [18]. CNN’s trained over large datasets, such as ImageNet, are widely used among the popular domains of computer vision, such as object recognition [19]. Transfer learning is used to utilize these trained networks over smaller datasets by fine-tuning the last fully connected layers. It has also proven to be robust for classification of cross-domain problems where CNN is trained over natural images and tested over the medical images [20].

In this paper, we utilize one of the CNN architectures for automatic analysis of brain MRI for Alzheimer’s prognosis. We evaluate the architecture AlexNet [21] by training it over the ImageNet dataset and classifying the multiple stages of Alzheimer’s from normal to mildly demented and moderately demented subjects using transfer learning. We also evaluate the effectiveness of utilizing the hierarchical features of natural images to classify medical imagery. The MRI scans acquired from the OASIS repository are tested under the proposed methodology covering the 3D views of the human brain. The proposed research work has the following main contributions:

- We propose and evaluate a transfer-learning-based method to classify Alzheimer’s disease
- An algorithm is proposed for a multiclass classification problem to identify Alzheimer’s stages
- We evaluate the effect of different gray levels 3D MRI views to identify the stages of Alzheimer’s disease

The rest of the paper is organized as follows. A literature review is presented in Section 2. Section 3 shows the proposed methodology, while Section 4 presents results followed by a conclusion.

## 2. Related Work

Over the last decade, many AD classification techniques have been proposed. We have categorized and analyzed these techniques based on the level of classification for both binary as well as multiclass classification.

### 2.1. Binary Classification Techniques

Beheshti et al. proposed a multi-staged model in a previous study [1], where he proposed a method for Alzheimer’s classification. The technique segmented the input images into GM, White Matter (WM), and Cerebral Spinal Fluid (CSF) as part of the pre-processing step. The technique utilized GM as a ROI to build the similarity matrices, through which the statistical features were extracted. Extracted features, along with the clinical data, were classified into AD and normal. Wang et al. [5] proposed a classification method for the test subjects based on the estimation of the 3D displacement field. Feature reduction was applied over the extracted features using feature selection techniques, such as Bhattacharya distance, student t-test, and Welch’s t-test. They then used the selected features to train the Support Vector Machine (SVM) classifier, classifying the test data with an accuracy of 93.05%.

Beheshti et al. in a previous study [1] devised a method to establish the significance of the volume reduction of GM. The method worked on the detection of shrinkage of GM both locally and globally using voxel-based morphometry (VBM). The regions that showed a significant reduction in the GM region were used for the segmentation of Volumes of Interest (VOI). VOIs were then used for the extraction of features and were optimized through a genetic algorithm. The optimized features were classified using SVM with an accuracy of 84.17%. Similarly, in a previous study [3] regions showing significant volume reduction in GM were selected as VOIs. They utilized the voxel values from the VOIs as the raw features that undergo feature reduction using feature ranking techniques. The selected features were then classified using SVM with an accuracy of 92.48%. Ramaniharan proposed an analysis of the variation in the shape of the corpus callosum followed by the segmentation of T1-weighted MRI scans [22]. It worked on the extraction of morphological features using the Laplace Beltrami eigenvalue shape descriptor. Reduced features based on the ranking of information gain were made part of the feature space, which was then classified using SVM and K-Nearest Neighbor (KNN). Out of the two classification algorithms, KNN outperformed SVM with an accuracy of 93.7%.

Guerrero proposed a feature extraction framework based on significant inter-subject variability [23]. ROIs were derived using a sparse regression model sampled for variable selection. An overall classification accuracy of 71% was achieved. Plocharski proposed a feature model based on the sulcal medical surface that classified Alzheimer’s patients against normal ones [24]. The features used were obtained from distinct patients and were classified with an accuracy of 87.9%. Ahmed et al. utilized circular harmonic functions (CHF), extracting local features from the regions of the hippocampus and posterior cingulate of the brain [25]. The classification of these extracted local features gave an overall accuracy of 62.07%. Use of deep learning was proposed by Sarraf in a previous study [17] using CNN. The CNN was utilized accompanied by auto-encoders, showing promising results with an accuracy of 98.4%.

### 2.2. Multi-Class Classification Techniques

A hybrid feature vector was formed incorporating cortical thickness along with the texture and shape of the hippocampus region [25]. It worked by classifying the feature vector combined with the MRI scans through a Linear Discriminant Analysis (LDA) classification algorithm. The methodology was tested over the images from the Alzheimer’s Disease Neuroimaging Initiative (ADNI) database over a multi-class classification problem giving an overall accuracy of 62.7%. Another multi-class classification technique was also proposed using the integrated features from the GM, WM, and CSF segments of the brain [26]. The extracted segments were then used to extract the statistical and textural features, classifying the samples as Alzheimer’s Disease AD, Mild Cognitive Impairment (MCI), or normal. The samples were obtained through ADNI [27] database and were classified with an accuracy of 79.8%. Looking at the techniques utilized in the literature, work has been done in both the binary and multi-class classification of Alzheimer. The techniques achieved promising results over the MRI images, however, they utilized conventional machine learning techniques for the Computer-Aided Diagnosis (CAD). A novel approach is proposed to accurately classify the 3D views of the brain MRI images using transfer learning.

### 2.3. Deep-Learning-Based Alzheimer’s Detection

In recent works, the use of deep learning methods has been significantly increased due to their high performance in comparison to conventional techniques. In a previous study [28], a hybrid method was devised comprising of extracted patches from an autoencoder in a combination of convolutional layers. An improvement was made in another study [29] by utilizing the 3D convolution. Autoencoders in the form of a stack led by a SoftMax layer were utilized in a previous study [12] for classification. The popular CNN architectures, such as LeNet, along with the first inception model were employed in a previous study [17]. Because of our smart selection of training data with the use of transfer learning, the use of a fractional number of training samples resulted in comparable accuracy with those being explored in medical imaging. A comparative analysis has been drawn in a previous study [30] providing an in-depth result of training a model from scratch versus fine-tuning. The analysis showed that in most of the cases, the latter performed better than the prior. Fine-tuned CNN has been utilized over multiple medical imaging problems, such as localizing planes in ultra-sound images [31], classification of interstitial lung diseases [20], and retrieval of missing plane views in cardiac images [32]. The use of transfer learning in the medical domain, as discussed above, proves its significance in achieving high accuracy in AD detection.

The existing work focuses on binary classification to identify a patient with Alzheimer’s disease. However, very little research work has been done to identify the stages of Alzheimer’s. In this work, we propose an efficient method that use 3D MRI images to identify the stages of Alzheimer’s disease.

## 3. Proposed Methodology

**Figure 1:**
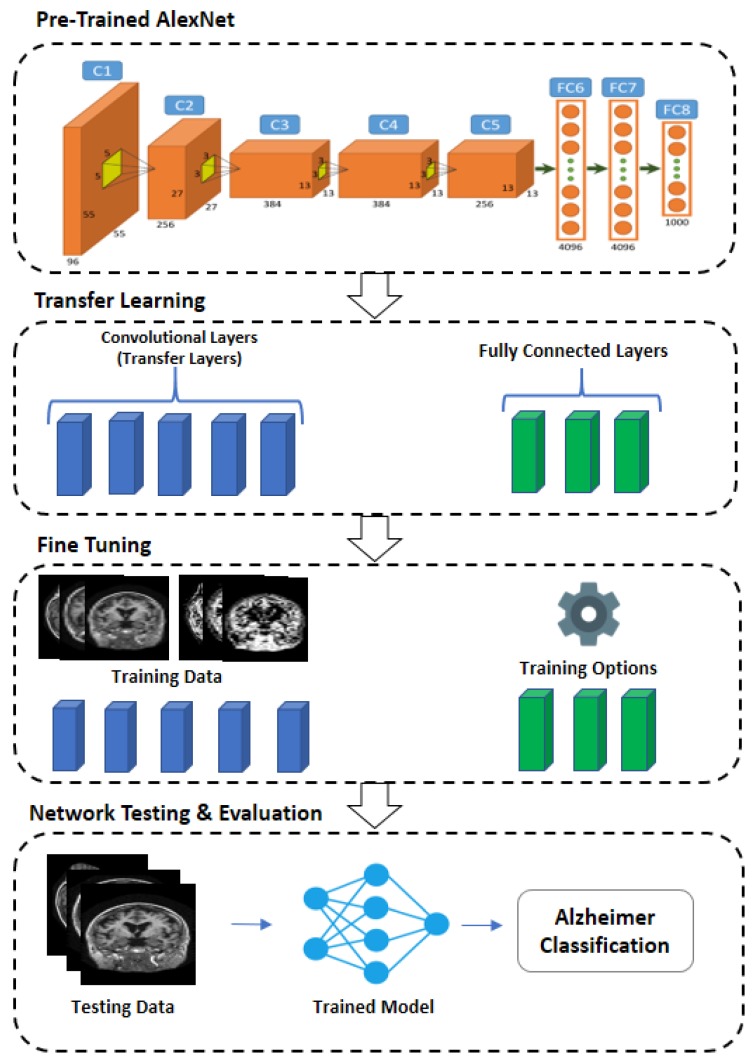
Proposed Methodology.

The proposed methodology exploits the transfer learning technique for Alzheimer’s detection. The key elements required to structure an effective CNN-based CAD model are discussed in the subsequent section. The proposed CNN-based Alzheimer’s detection is shown in Figure 1.

### 3.1. Pre-trained CNN Architecture: AlexNet

A CNN is a specialized form of multi-layered neural network that is based on the pattern recognition from image pixels directly, with minimum pre-processing required [33]. A typical CNN is comprised of three basic layers, given as the convolution layer, pooling layer, and fully connected layer. The convolution layer is the core building layer of a CNN and is responsible for most of the computational work done. As its name indicates, it performs the convolution operation or filtration over the input, forwarding the response to the next layer. The applied filter acts as a feature identifier, filtering the entire input and forming a feature map. In between the consecutive convolution there is the pooling layer, used to spatially reduce the spatial representation and the computational space. The pooling layer performs the pooling operation on each of the sliced inputs, reducing the computational cost for the next convolution layer. The application of convolution and pooling layers results in the extraction and reduction of features from the input images. The final output equal to the number of classes is generated by applying the fully connected layer. A CNN is a stacked version of all these layers forming a CNN architecture. Each CNN follows the same architecture, however, with a few variations. In this work, we have utilized the AlexNet pre-trained architecture for Alzheimer’s detection [34].

### 3.2. Transfer Learning Parameters

The transfer learning concept is commonly used in deep learning applications. The transfer learning approach is helpful if we have a small training dataset for parameter learning. We take a trained network, e.g., AlexNet as a starting point to learn a new task. AlexNet is trained on a large dataset of ImageNet with a large number of labeled images. We transfer the pre-trained parameters of internal layers of AlexNet (all layers except last three layers) and we replace the three fully connected layers of AlexNet with the softmax layer, fully connected layer, and output classification layer.

A domain D consists of two components: feature space represented by *X*, and its respective marginal probability *P*(*X*) [35], where *X* = {*x*_1_, *x*_2_, …, *x*_n_} and n is the number of input images. Mathematically, a domain can be represented as

(1) D={ X, P(X)}

For two different domains, their respective feature space along with their marginal probabilities would also be different. In a domain D, a task T is also represented by two components, label space *Y* and objective predictive function f(.). Mathematically, this is given as

(2) T={Y, f(.)}

The predictive function *f*(.) is learned during the training process of features *X* labeled as *Y* and is used to predict the testing data. In our proposed method of utilizing a pre-trained network of AlexNet, we experience the case where there is one source domain *D_s_* and one target domain *D_t_*. The source domain data is denoted as

(3) $$D_{s}=\{\;\left(x_{s1},y_{s1}\right),\;\left(x_{s2},\;y_{s2}\right)\ldots \ldots \ldots .\;\left(x_{sn},\;y_{sn\;}\right)\}$$

where x_si_ is the source data instance having a corresponding label y_si_. Similarly, the target domain data is also represented as

(4) $$D_{t}=\left\{\left(x_{t1},\;y_{t1}\right),\;\left(x_{t2},\;y_{t2}\right)\ldots \ldots \ldots \ldots \left(x_{tn},\;y_{tn}\right)\right\}$$

where *x_ti_* is the target instance and *y_ti_* is the target label. In our proposed system the two domains and their respective feature space and labels are not the same. Transfer learning is the learning of the predictive function *f*(.) of the target domain using the knowledge acquired from the source domain and source tasks; *f*(.) is utilized to predict the label *f*(*x*) of the new instance *x*, where *f*(*x*) is mathematically represented as

(5) f(x)=P(y|x)

In conventional machine learning techniques, *D_s_* = *D_t_*, and similarly the *T_s_* = *T_t_*. In our proposed methodology the source and the target domain are dissimilar, causing the components to be dissimilar as well, implying either *X_s_* ≠ *X_t_* or *P*(*X_s_*) ≠ *P*(*X_t_*). As for the tasks T, the label *y_t_* and *y_s_* are also not equal. AlexNet is a pre-trained CNN over the natural images dataset ImageNet forming the source Domain *D_s_*. The CNN architecture of AlexNet contains in excess of 60 million parameters. Taking in such huge numbers of parameters from just a thousand training images in a straightforward manner is dangerous. The key idea of this work is that the inside layers of the CNN can extract generic features of images, which can be pre-prepared in one domain *D_s_* (the source errand, here ImageNet) and then these parameters can be re-utilized for classification of a new task *D_t_* (Alzheimer’s classification).

#### Modified Network Architecture

**Figure 2:**
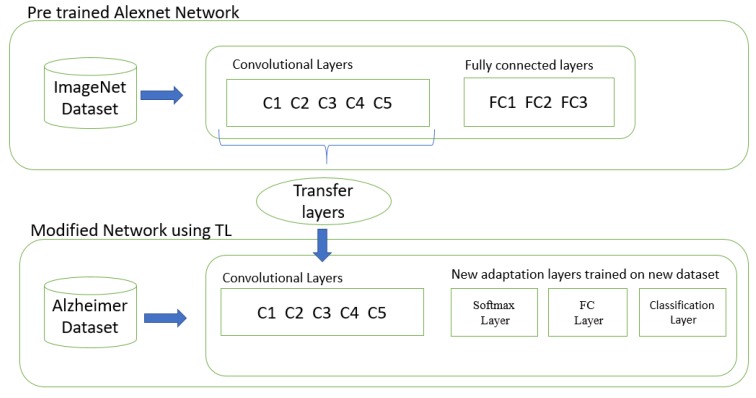
Transferring CNN layers.

We use the pre-trained architecture AlexNet. The system takes a square 227 × 227-pixel RGB picture as the input and disseminates this over the ImageNet protest classes. This system is made from five progressive Convolutional (C) layers C1–C5 pursued by three completely associated Fully Connected (FC) layers FC6–FC8 (Figure 2).

For new tasks (Alzheimer’s classification) we wish to outline a system that will produce scores for target classes. It is possible that the classes learned by the pre-trained network vary from the classes of the new task. The early layers of the network extract the generic features of training images, such as edge detection, while the last fully connected layers learn the class-specific features to categorize the images into specific classes. In order to achieve the transfer learning, we extract all the layers of AlexNet (except last three layers) as transfer layers and replace the last three layers of AlexNet with modified SoftMax layers, fully connected layers, and an output classification layer, so that they learn the class specific features of the Alzheimer’s dataset.

The first 5 layers are trained using AlexNet (ImageNet) and remain fixed, while Alzheimer’s datasets are used to train the remaining adaptation layers. The parameters used for the fully connected FC layer are biased learn factor, weight learn factor, and the size of the output. The output size of the FC layers is set equal to the class labels. The bias learning rate depends upon the bias learn factor, while weight learn rate handles the learning rate and a value of 50 is used for both bias learning rate and learning rate. Softmax functions are applied to the input by the Softmax layer. Classification parameters include output size and function name utilized for the multi-class classification. Cross-Entropy Function for k Mutually Exclusive Classes (cross entropy) was employed as the loss function and the number of classes determined the output size. 

### 3.3. Pre-Processing of Target Dataset

Prior to the training and testing of image samples from the target domain, they undergo the process of pre-processing. MRI scans during the process of their formation may undergo degradation, such as low contrast, due to bad luminance caused by the optical devices. To improve the visual characteristics of the images, image enhancement techniques are formed, such as linear contrast stretching, to improve the distribution of pixels over a wider range of intensities. A pixel-based operation is performed mapping each input pixel value to its corresponding output intensity value. For an input pixel with intensity x in an image with minimum intensity level b and maximum a, this is mapped over the output pixel with intensity level *y*, forming an enhanced image with c as the maximum grey level, which is given in the equation below.

(6) $$y=c\left(\frac{x-b}{a-b}\right)$$

### 3.4. Training Network and Fine Tuning

The AlexNet architecture is trained over the ImageNet dataset comprised of images belonging to 1000 classes. To train the pre-trained model for the classification of images from the target domain, the transferred CNN layers are finely tuned over the target dataset, keeping the low-level features from ImageNet intact. CNN architecture is trained over the target domain with a quick learning rate and the class distinctive features are incorporated in the layers to be finely tuned for the target domain classification. The last three fully connected layers are configured for the target domain, allowing them to classify the target images into their respective number of classes. The motivation behind the process is to transfer the low features in the shallow layers of the model to be used for the problem domain, speeding up the learning rate for a new problem.

The proposed method was trained using 40%, 50%, and 60% of the total samples. There are some parameters that are used to train the network or a training option can be used for the network. The training option includes Batch Size, Number of Epochs, Learning rate, and validation frequency. To train the network we use a Batch size of 10 and 1e−4 learning rate. The maximum number of epochs used for training is 10, while the bias learn factor and weight learn factor are both 50 [34]. The optimization algorithm used for training is Stochastic Gradient Descent with Momentum (SGDM). This optimization algorithm minimizes the loss function and adjusts the bias and weight parameter. The new adaptation layers learn the features of the Alzheimer’s dataset using these training options.

### 3.5. Network Testing—Classification of Alzheimer’s

The Monte Carlo method was used for 100 simulations under the following system settings. We varied the parameters (learning rate, number of epochs, learn rate factor, and bias learn factor) to fine tune the network. An algorithm for transfer learning was applied on 6, 10, 15, 20, and 25 epochs and found that the optimal number of epochs is 10. We checked the performance by changing the learn rate from 1e−1 to −1e−10, weight learn factor and bias learn factor from 10–100, MiniBatchSize size 10 to 90, and found that optimal results were obtained on 1e−4 learning rate, learn and bias learn factors of 50, and MiniBatchSize of 10. We tested the network by varying the testing and training data. The testing set comprised of 40%, 50%, and 60% data of the original sample and ran the simulations by varying the testing data. Finally, we averaged the results of every testing data.

It was found that the application of AlexNet (pre-trained upon the ImageNet dataset) over the dataset of medical images forming target domain *D_t_* showed promising results. Apart from the dissimilarity between the natural images of the source domain and to that of the medical images of the target domain, transferring knowledge of large datasets of natural images contributes to the effectiveness of Alzheimer’s detection. 

## 4. Experimental Setup and Results

### 4.1. Tools and Software

Our proposed methodology employed a convolutional neural network architecture for the classification of Alzheimer’s disease in patients. It is a multi-step-based algorithm executed on an HP Core i5-8400. All the simulations were performed in MATLAB 2018.

### 4.2. Dataset

CNN parameters were initialized using the ImageNet classification, referred to as pre-training. The AlexNet architecture is pre-trained over the large dataset of ImageNet of over 1.2 million labeled high-resolution images. The images belong to about 1000 categories collected over the web and labeled by human labelers. The process of adapting to the pre-trained CNN to train over the target dataset is referred to as fine-tuning. The target dataset is taken from the publicly available OASIS repository [36] of brain MRI scans of normal, very mildly demented, mildly demented, and Alzheimer’s patients. The dataset provided the cross-sectional brain MRI scans covering multiple sagittal, coronal, and axial views. A total of 382 image samples were taken belonging to subjects of ages from 18 to 96, covering the progression of Alzheimer’s at each age level. The model was trained over the dataset of whole images along with the segments of Grey Matter, WM, and Cerebral Spinal Fluid. The images in the dataset were accompanied by their respective CDR values serving as the ground truth data listed in the table below. Both the training and testing samples were ensured to cover all the stages of AD characterized by the respective CDR values, as given in Table 1. 

### 4.3. Image Pre-Processing

Noise is likely to be added during the process of image acquisition. Any unwanted information added to the image due to either motion blur or non-linear light intensity is considered as noise. When it comes to medical imaging, non-linear light intensity majorly affects the overall performance of the image processing [34]. Non-linearity of light is mainly introduced due to the false setting of the lens apertures of capturing devices. The uneven distribution of light can be normalized by image enhancement techniques. Contrast stretching is used to expand the dynamic range of light intensity, resulting in an image with better contrast and light distribution [37]. Images in the OASIS repository were enhanced using the linear contrast stretching for improved performance in the latter stages.

### 4.4. Image Segmentation

MRI scans taken from the OASIS repository consist of images capturing the internal structure of the human brain. The images are segmented by extracting the varying intensities of GM, WM, and CSF in the captured brain information [38]. These segments are extracted using the K-Mean clustering, dividing the image into non-overlapping regions. The proposed method is evaluated over MRI scans from the OASIS repository along with the segmented components. Both whole and segmented images are re-sized to 227 × 277 as per required configuration. These pre-processed images are fed into the fully connected layers of the AlexNet model, upon which it is finely tuned over the target domain.

### 4.5. Evaluation Metrics

Classification results obtained through the AlexNet architecture are evaluated using different evaluation metrics [39]. Each metric is briefly discussed below.

#### 4.5.1. Sensitivity–Recall

This is defined as the ratio of truly positive predicted instances to all the positive instances in ground information. It gives the performance of the classification of positive labeled instances. Mathematically, it is given as

(7) $$Sensitivity=\frac{TP}{TP+FN}$$

#### 4.5.2. Specificity

This is defined as the ratio of truly negative predicted instances to all the negative instances in the ground information. It gives the performance of the classification of negative labeled instances. Mathematically, it is given as

(8) $$Specificity=\frac{TN}{TN+FP}$$

#### 4.5.3. Precision

This is defined as the ratio of correct prediction of truly positive instances among all the instances that were classified as the positive ones [33,36,40]. Mathematically, it is given as

(9) $$Precision=\frac{TP}{TP+FP}$$

#### 4.5.4. False Positive Rate (FPR)

This is also known as the false alarm rate and is defined as the ratio of falsely positive predicted instances to the true negative instances. It is also known as type II error, and is mathematically given as

(10) $$FPR=\frac{FP}{TN+FP}$$

#### 4.5.5. Equal Error Rate (EER)

This is defined as the intersecting point between the plotted graphs of False Positive Rate and False Negative Rate. It is the representation of the point where the mentioned two metrics become equal. EER is the point where the false acceptance and false rejection become equal and optima, and mathematically it is given as

(11) $$EER=\;\frac{FP}{TN+FP}=\;\frac{FN}{TP+FN}$$

In the above equations, FP, FN, TP, TN are given as false positives, false negatives, true positives, and true negatives, respectively. 

### 4.6. Results and Discussion 

#### 4.6.1. Pre-Processing Results

Linear contrast stretching improves the intensity distribution of the image by expanding the overall distribution of image information represented by a histogram. MRI scans may require pre-processing to reduce the non-linearity of light added due to the false configuration of the capturing devices. 

**Figure 3:**
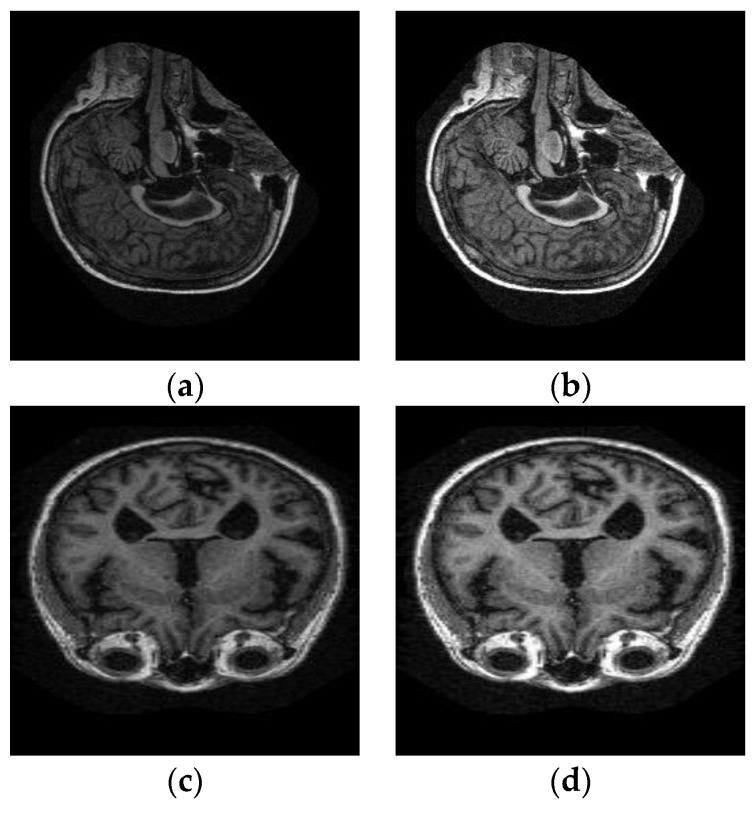
(**a**,**c**) Input MRI scans. (**b**,**d**) Contrast Stretched output Images.

**Figure 4:**
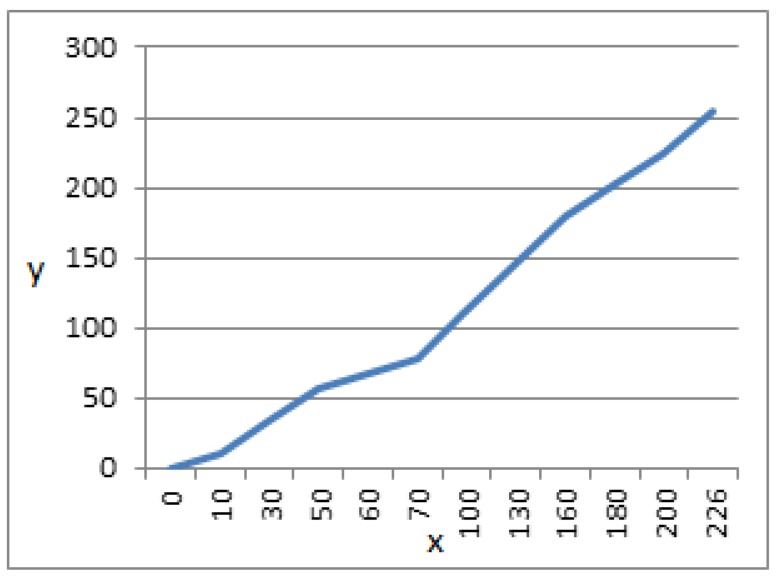
Linear Contrast Stretching for y=1.128x.

The input image pixel intensities are mapped to a wider range of intensity values, stretching them to the extreme limits. This adds contrast in the image, highlighting the MRI scan on a dark background. Results of linear contrast stretched brain MRIs are shown in Figure 3. The relationship between the input intensity distribution and output intensity distribution is given in Figure 4.

#### 4.6.2. Segmentation Results

**Figure 5:**
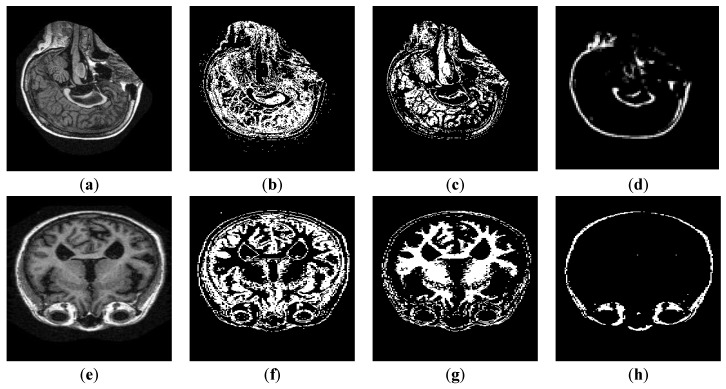
(**a**,**e**) Input-enhanced MRI. (**b**,**f**) GM segment of input MRI. (**c**,**g**) WM segment of input MRI. (**d**,**h**) CSF segment of input MRI.

Pre-processed MRI scans from the OASIS repository were segmented using K-Mean clustering with a K value of 4. The varying intensities of the brain scans were utilized to extract the internal components of GM, WM, and CSF. The pre-processing stage stretches the range of the intensity values, increasing the difference among the varying intensities of the brain components. These individual segmented components form the internal compartmental model of the human brain. The enhanced brain MRI scan along with the respective segments are shown in Figure 5. 

#### 4.6.3. Layer-Wise Results of AlexNet

**Figure 6:**
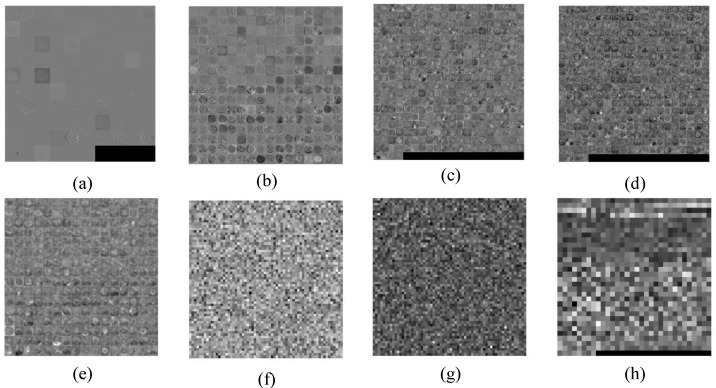
(**a**–**e**) Feature extraction results of convolutional layers C1, C2, C3, C4, and C5. (**f**–**h**) Feature extraction results of fully connected layers FC6, FC7, and FC8.

The pre-processed segmented and un-segmented MRIs were then passed into the pre-trained AlexNet architecture. The low-level features from the ImageNet dataset are retained in the lower layers of the convolutional architecture and are transferred during the training of the network over the target domain. Features are extracted at each layer, retaining the low-level features of the pre-trained architecture and fine-tuning them over the target domain. Layer-wise results of the features of the finely tuned AlexNet architecture are shown in Figure 6. Results in Figure 6a–h represent the features extracted from the first convolutional layer to last fully connected layer.

Figure 6a shows the features extracted by the convolutional layer1 (C1) if trained on the target domain. Similarly, Figure 6b–e represents the visual representation of features by the convolutional layer (C2, C3, C4, and C5, respectively). We observe that the convolutional layer represents the generic features of the images representing the edge detection of images, while Figure 6f–h represents the features extracted by fully connected layers (FC6, FC7, and FC8, respectively) of AleNxNet. We observe that fully connected layers learn class-specific features to distinguish among classes. Therefore, AlexNet is trained on thousands of classes, meaning features are not prominent due to many domain classes. In our target dataset, we have fewer classes, so we modified the fully connected layers to learn the target class-specific features instead of domain class-specific features.

#### 4.6.4. Classification Results

**Figure 7:**
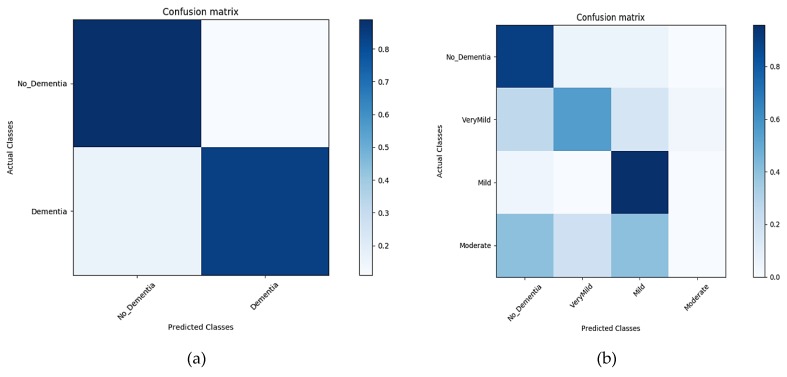
The confusion matrix of the Segment1 dataset (**a**), representing the confusion matrix of the binary dataset. (**b**) A confusion matrix of multiple classes.

Images from the OASIS repository (whole along with the segmented) are classified through the CNN architecture using transfer learning for both the binary and multi-class problem. Alzheimer’s detection is of high significance for research in medical sciences, however, the diagnosis of its stages serves as an aid for its in-time treatment, thus increasing the significance of the multi-class problem even more. Shallow convolutional layers of the AlexNet model pre-trained over the ImageNet dataset are transferred, comprised of the low-level features extracted from over 1,000,000 images. With the convolutional layers being transferred, the layers in the AlexNet architecture are finely tuned over the whole and segmented brain scans. The last three layers are then configured for training options for the new classification problem. The segments of GM, WM, and CSF, along with the un-segmented image, form the four datasets upon which the CNN model is trained to learn the task-specific features. The pre-trained model AlexNet has the convolutional layers transferred, keeping the low-level features from the source domain, thus speeding up the learning rate. Training and testing processes for each dataset were recorded over a time period of 10 epochs and their corresponding evaluation results in the form of the confusion matrix were obtained. The proposed method is trained and tested for both binary and multi-class Alzheimer’s classification. Training and testing in the form of confusion matrices are shown in Figure 7. The results show the classification results over the segmented MRIs, with GM, WM, and CSF as the individual segments. 

##### Classification Results for GM—Segment 1

The results in Figure 7a,b show the classification accuracy in the form of the confusion matrix generated during the classification of the first segment (GM) for the time interval of 10 epochs for both binary and multi-level classification.

##### Classification Results for WM—Segment 2

**Figure 8:**
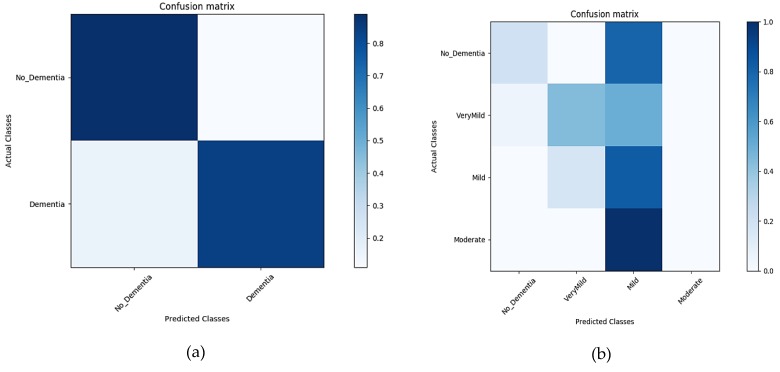
Confusion matrix of the Segment2 dataset (**a**), representing the confusion matrix of the binary dataset. (**b**) A confusion matrix of multiple classes.

Another important component of the internal composition of the brain is the WM and is considered as the second segment to classify. The results in Figure 8a,b below show the classification accuracy in the form of the confusion matrix generated during the classification of the second segment (WM) for the time interval of 10 epochs for both binary and multi-level classification. 

##### Classification Results for CSF—Segment 3

**Figure 9:**
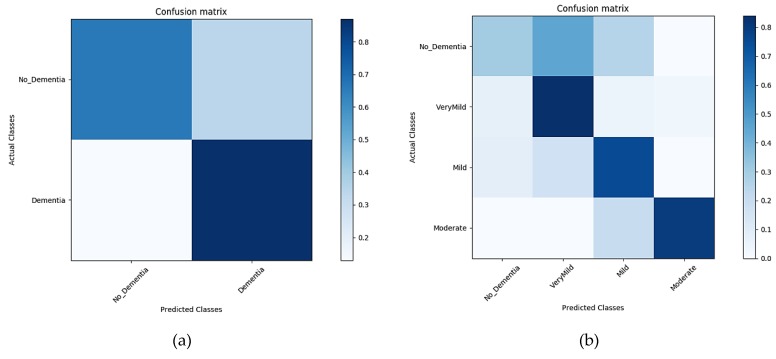
Confusion matrix of the Segment 3 dataset (**a**), representing the confusion matrix of the binary dataset. (**b**) A confusion matrix of multiple classes.

The liquid matter surrounding the brain and spinal cord is the CSF. Likewise, GM and WM is another important component of the human brain. With GM as segment 1 and WM as segment 2, CSF constituted the third segment of the human brain. The results in Figure 9a,b below show the classification accuracy in the form of the confusion matrix generated during the classification of CSF for the time interval of 10 epochs for both binary and multi-level classification.

##### Classification Results for Un-Segmented MRIs

**Figure 10:**
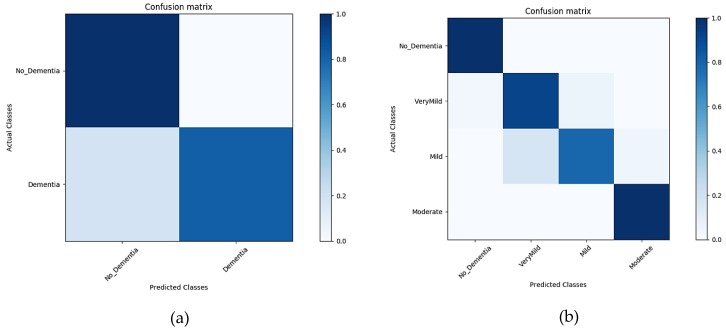
Confusion matrix of un-segmented images (**a**), representing the confusion matrix of a binary dataset. (**b**) A confusion matrix of multiple classes.

GM, WM, and CSF incorporated into a single image constituted the original MRI. The input MRIs carried cumulative information of all the brain components in a single image. The results in Figure 10a,b below show the classification accuracy in the form of confusion matrix generated during the classification of the un-segmented MRI for the time interval of 10 epochs for both binary and multi-level classification. 

**Figure 11:**
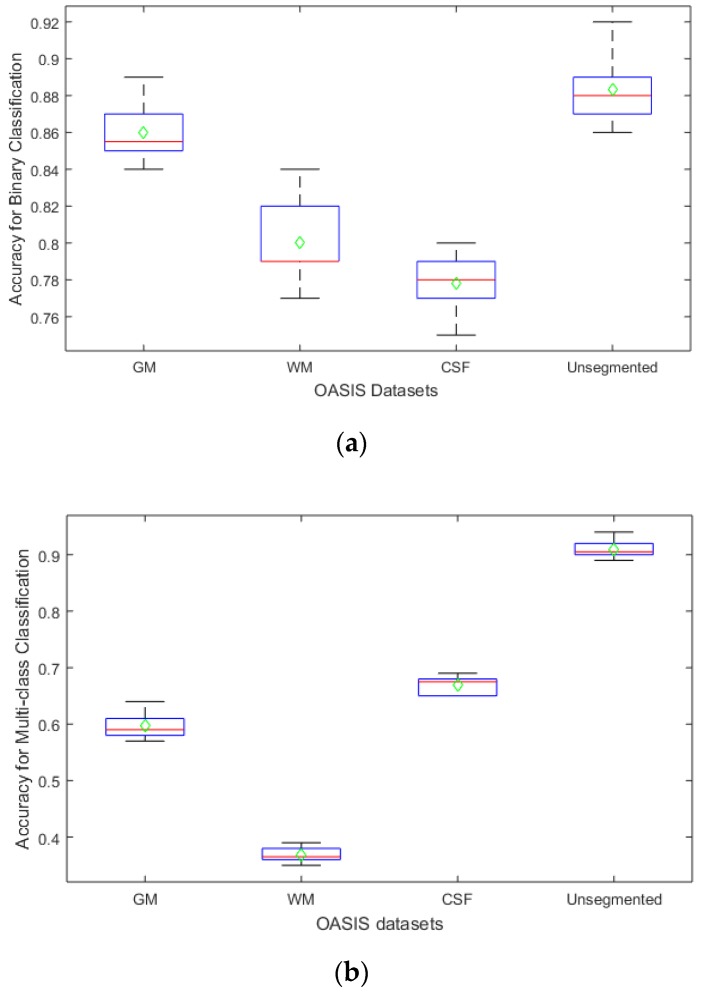
Boxplot of the results on OASIS dataset under proposed segmentation—GM, WM, CSF, and Un-segmented, in terms of accuracy. The arithmetic mean is presented in green and the median in red (**a**) represents the results for binary dataset. (**b**) Classification results of multiple classes.

The Monte Carlo method was employed for 100 simulations, in which we varied our parameters, i.e., learning rate, number of epochs, learn rate factor, and bias learn factor, to fine-tune our network. Using the Monte Carlo method, the accuracy of our algorithm was reported for varying parameters using their optimal values. Box and whisker plots of our proposed methods for both binary and multi-class classification are given in Figure 11 below.

Using the Monte Carlo method, average classification accuracies, represented by green diamonds, were obtained under optimal parameter values of 10, 1−e4, and 50 for the number of epochs, learning rate, and learn and bias factors, respectively. 

Table 2 represents a closer analysis of the varying number of epochs (6, 10, 15, 20, and 25) with varying classification results. We observed that average performance obtained at 10 epochs outperformed the performances of other scenarios. When we increase the epochs the accuracy decreased.

The evaluation metrics are extracted from the confusion matrices, presenting the overall performance of the CNN models over transfer learning-based classification of MRI scans for both binary and multi-class classification problems. The extracted evaluation metrics, as discussed in the previous section, are listed below in Table 3. 

From the statistical analysis of the classification accuracies and estimation errors, it has been observed that the system performed at its best for multi-class classification of un-segmented images. It can be deduced from the results that the cumulative information of GM, WM, and CSF proved to be distinctive enough for the better classification of the AD. The un-segmented images as inputs reduced the computational complexity by two-thirds and resulted in high accuracy of 92.8%. The learning time for each classification scenario is given in Table 3. These learning times are highly dependent on machine specifications. Alzheimer’s detection and classification are of high significance for both binary and multi-class problem among researchers. Work has been done in both binary and multi-class classification of Alzheimer’s, as given in the related work section. A comparative analysis of the proposed methodology has been made with the existing techniques that handle multiclass classification. 

Considering the techniques proposed in the past, high accuracy has been achieved in classifying the test subjects as having Alzheimer’s or not. However, transfer learning has never been used to test the accuracy of the system, which serves as a novel approach towards Alzheimer’s classification. With high accuracy achieved in the diagnosis of Alzheimer’s in a test subject, detection of the level of progression is of high significance. The spread of disease allows the treatment required for the patient to be determined. A few instances of implementation can be seen in the past, however, none of these used the proposed methods. 

A hybrid feature vector of textural and clinical data was utilized by Tooba at al. for multi-class classification of Alzheimer’s in the sample, using MRI scans. The technique achieved an overall accuracy of 79.8% for multi-class classification. Similarly, another hybrid feature vector was also classified, formed by the combination of structural and morphological features. Sørensen et al. classified the hybrid feature vector for multi-class classification, resulting in an accuracy of 62.7%. In comparison to the techniques discussed above, our proposed method of utilizing AlexNet architecture for transfer learning achieved the highest accuracy of 92.8%, as shown in Table 4. 

## 5. Conclusions

The detection of Alzheimer’s stages remains a difficult problem because of the multiclass classification task. In this work, we proposed an efficient and automated system based on a transfer learning classification model of Alzheimer’s disease for both binary and multi-class problems (Alzheimer’s stage detection). The algorithm utilized a pre-trained network, AlexNet, and fine-tuned the CNN for our proposed problem. The proposed model was fine-tuned over both segmented and un-segmented 3D views of human brain MRI scans. The MRI scans were segmented into characteristic components of GM, WM, and CSF. The re-trained CNN was then validated using the testing data, giving overall accuracies of 89.6% and 92.8% for binary and multi-class problems, respectively. It was observed that the unsegmented images carried enough information to be accurately classified in comparison to the segmented scans. In our future work, we will analyze the accuracy of the system by fine-tuning all of the convolutional layers and will explore the effectiveness of other state-of-the-art CNN architectures. 

## Figures and Tables

**Table 1:** Clinical Dementia Result (CDR) values with the corresponding mental state in the OASIS dataset.

Clinical Dementia Rate (CDR)	Corresponding Mental State	No. of Image Samples
0	No Dementia	167
0.5	Very Mild Dementia	87
1	Mild Dementia	105
2	Moderate Dementia	23

**Table 2:** Results of transfer learning for different numbers of Epochs.

Dataset	Classification	6 Epochs	10 Epochs	15 Epochs	20 Epochs	25 Epochs
OASIS (Un-segmented)	Binary	0.84	0.89	0.89	0.83	0.85
	Multiple	0.86	0.92	0.91	0.87	0.87

**Table 3:** Results of transfer learning on four datasets.

Dataset	Classification	Sensitivity	Specificity	Precision	EER	FPR	F1 Score	Accuracy	Learning Time
OASIS (GM)	Binary	0.89	0.84	0.84	0.13	0.16	-	0.8621	93 min 6 s
	Multiple	60.28%	49.20%	49.20%	0.40	0.51	54.18%	0.6028	76 min 14 s
OASIS (WM)	Binary	0.66	0.92	0.89	0.2	0.08	-	0.8046	120 min 4 s
	Multiple	37.01%	35.25%	35.25%	0.63	0.65	36.11%	0.3701	113 min 23 s
OASIS (CSF)	Binary	0.66	0.88	0.85	0.22	0.12	-	0.7816	118 min 13 s
	Multiple	67.33%	62.63%	62.63%	0.33	0.38	64.90	0.6733	110 min 24 s
OASIS (Un-segmented)	Binary	1	0.82	0.847	0.1	0.18	-	0.8966	125 min 20 s
	Multiple	92.85%	74.27%	74.27%	0.07	0.26	82.53	0.9285	117 min 15 s

**Table 4:** Comparative Analysis with Multi-class Classification Techniques.

Sr#	Algorithm	Classifier	Accuracy (%)
1	Proposed Algorithm	CNN	92.8
2	MRI + clinical data	SVM	79.8
3	MRI + hybrid feature	LDA	62.7
